# An Unusual Presentation of Extra-Oral Plasmablastic Lymphoma With Unique Cytomorphology

**DOI:** 10.7759/cureus.16562

**Published:** 2021-07-22

**Authors:** Priyadarshini Dehuri, Debahuti Mohapatra, Prateek Das

**Affiliations:** 1 Pathology, Institute of Medical Sciences and SUM Hospital, Bhubaneswar, IND

**Keywords:** plasmablastic lymphoma, immunocompetent, gastrointestinal, cytomorphology, non hodgkin lymphoma

## Abstract

Plasmablastic lymphomas are high-grade lymphomas most commonly observed in the oral cavity. Their association with HIV-infected patients is now well-known. The occurrence of plasmablastic lymphomas in extra-oral sites in immunocompetent patients is exceedingly rare. We aim to document such a rare case in a 69-year- old female in the gastrointestinal tract along with lymphomatous effusion of the pleural cavity. The discussed case also needs a mention for its unique cytomorphological features. The diagnosis was confirmed by immunohistochemical stains, which play a vital role in the accurate diagnosis of plasmablastic lymphomas and their distinction from other anaplastic non-Hodgkin lymphomas.

## Introduction

Plasmablastic lymphoma is a relatively new entity that has grabbed attention in the last two decades since 1997 when it was first categorized by Delecluse et al. [1]. They concluded that it was a lymphoma highly associated with HIV infection and commonly seen in the oral cavity. In the subsequent years, there have been reports of this entity in oral as well as extraoral sites [2]. However gastrointestinal plasmablastic lymphomas have been documented in few case reports and two case series only [3-6]. We present a case of gastric plasmablastic lymphoma in a patient with malignant lymphomatous effusion of the pleural cavity. The cytomorphological features of this case also stand out, far from the typical plasmablastic or recently described plasmacytic features.

## Case presentation

**Figure 1:**
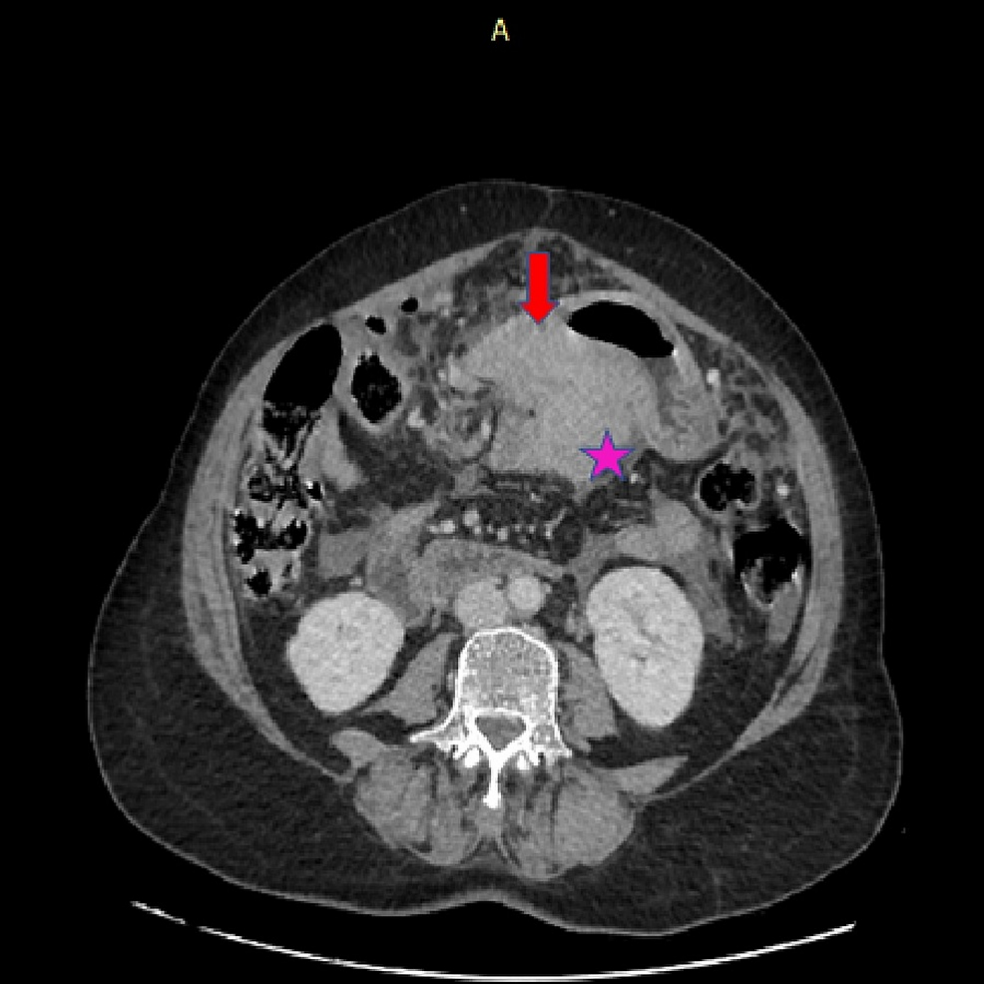
Image of axial section of CECT abdomen showing gastric wall thickening in the antropyloric region (red arrow) with enlarged perigastric lymph nodes (pink star) CECT - contrast-enhanced computed tomography

**Figure 2:**
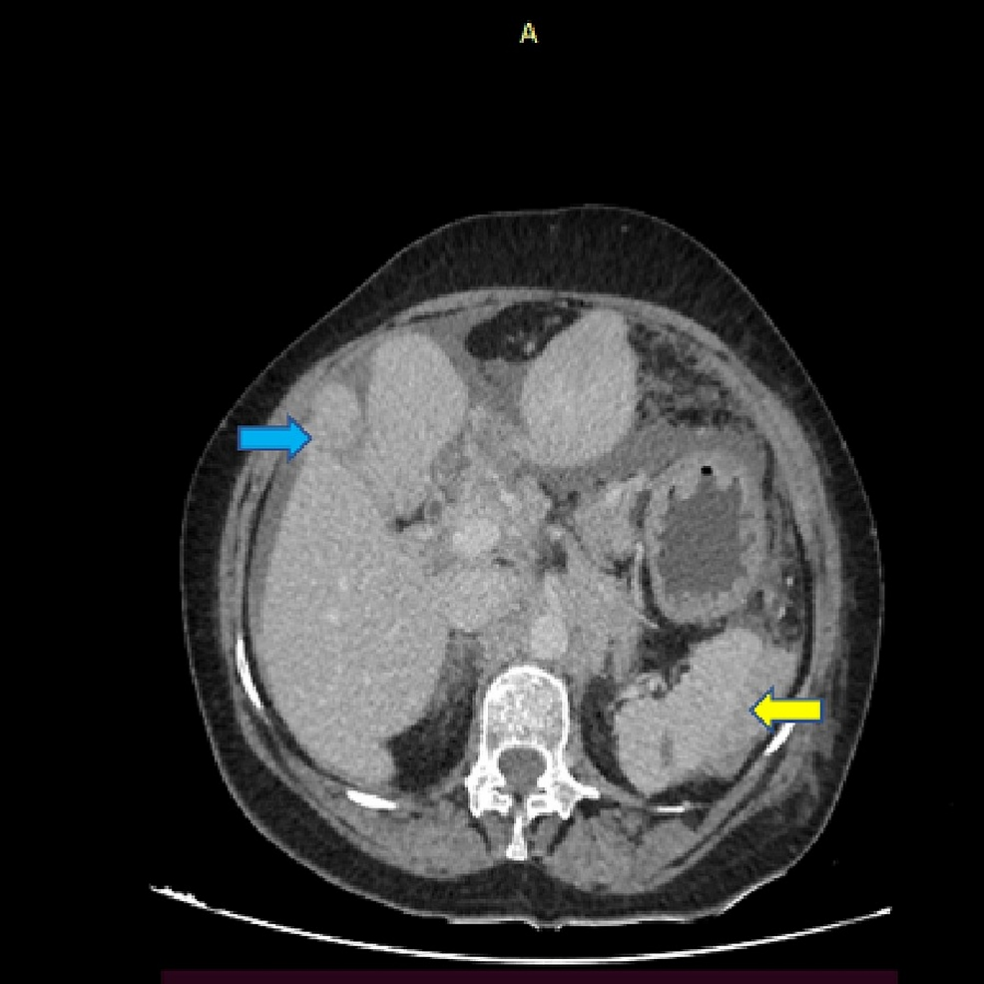
Image of axial section of CECT abdomen showing surface deposits over liver parenchyma (blue arrow) and spleen (yellow arrow) CECT - contrast-enhanced computed tomography

A 69-year-old female presented with chest pain, loss of appetite, and abdominal pain. Upper gastrointestinal endoscopy showed a patch of indurated mucosa of size 10 mm x 10 mm over the proximal body of the stomach along the lesser curvature. An excavated ulcer of size 30 mm x 30 mm was noted in the distal part of the body in the anterior wall along the lesser curvature with edematous adjacent mucosa. The margins of the ulcer appeared rolled out. The first part of the duodenum showed ulceroinfiltrated mucosa with a sloughy base. The second part of the duodenum appeared unremarkable. Contrast-enhanced computed tomography (CECT) of the abdomen showed diffuse circumferential thickening of the body and antropyloric region of the stomach (Figure 1). Multiple conglomerated necrotic lymph nodes, the largest measuring 4.3x3.2 cm, were seen in the lesser sac region (Figure 1). Numerous irregular hypoechoic deposits over the hepatic and splenic surfaces were observed (Figure 2). Based on these findings, gastric carcinoma with nodal spread was suspected. CT chest showed bilateral mild pleural effusion with the collapse of the basal segments. Pleural fluid was aspirated and sent for cytological study. The available hematological and serum biochemical parameters were within normal limits (Table 1). She did not have a history of intake of any steroids or other drugs. Her retroviral serology was negative. There were no known comorbidities either.

**Table 1:** Laboratory investigations SGOT - serum glutamic oxaloacetic transaminase; SGPT - serum glutamic pyruvic transaminase; LDH - lactate dehydrogenase; ADA - adenosine deaminase; HBV - hepatitis B virus; HCV - hepatitis C virus

Serum bilirubin (Total)	0.94 mg/dl
Serum Bilirubin (Direct)	0.26 mg/dl
Serum transaminases (SGOT)	25.3 IU/L
Serum transaminases (SGPT)	19.5 IU/L
Total protein	8 gm/dl
C-reactive protein	0.1 mg/dl
Pleural fluid LDH	423U/L
Pleural fluid ADA	18 U/L
Triple H status (HIV, HBV, HCV)	Negative

**Figure 3:**
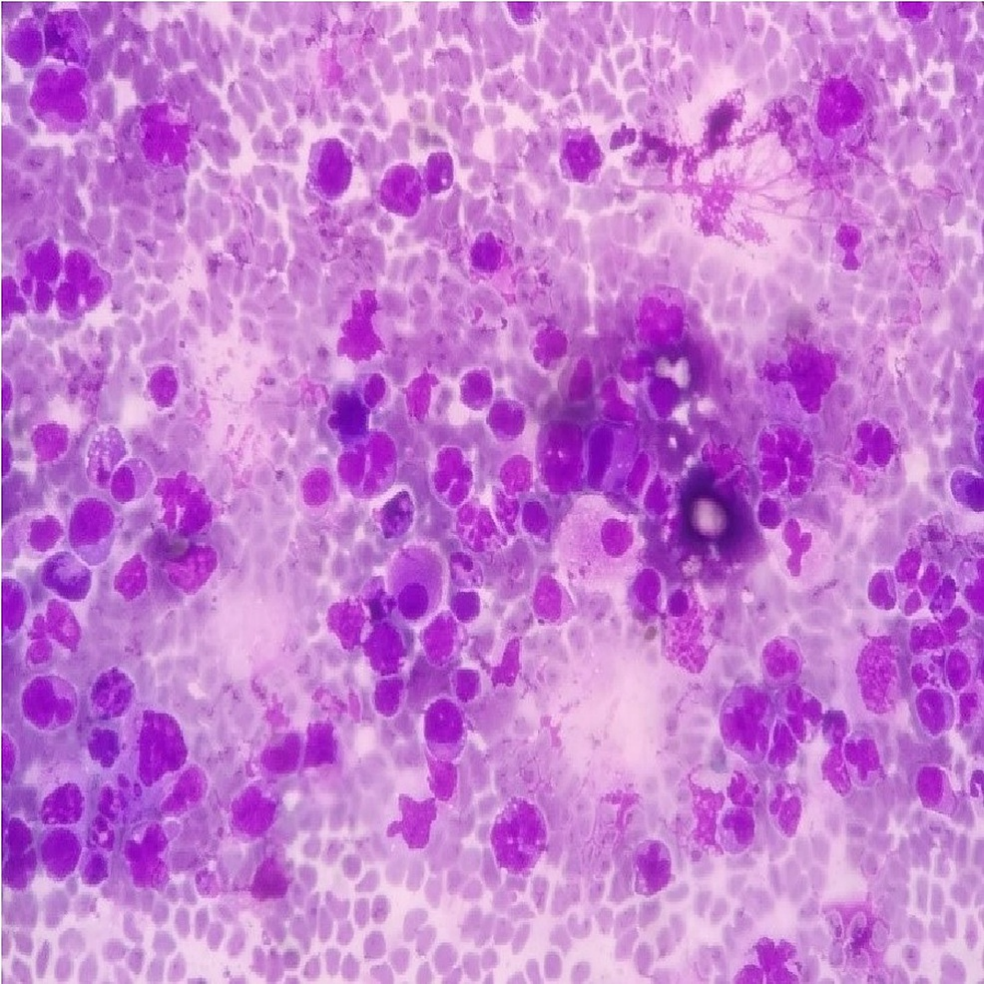
x400 Diffquik stain of cytology smear showing abnormal lymphoid cells with varied morphology

The pleural fluid cytological smears (both wet-fixed and air-dried) showed atypical lymphoid cells of varied morphology, including plasmacytoid cells with reactive mesothelial cells and lymphocytes in the background. There were cells with a moderate amount of cytoplasm and indented and multilobulated nuclei (Figure 3). A good number of apoptotic bodies and mitotic figures were noted. The cytological examination was interpreted as lymphomatous effusion with advice for flow cytometric analysis of the fluid. However, the test could not be performed due to financial constraints.

**Figure 4:**
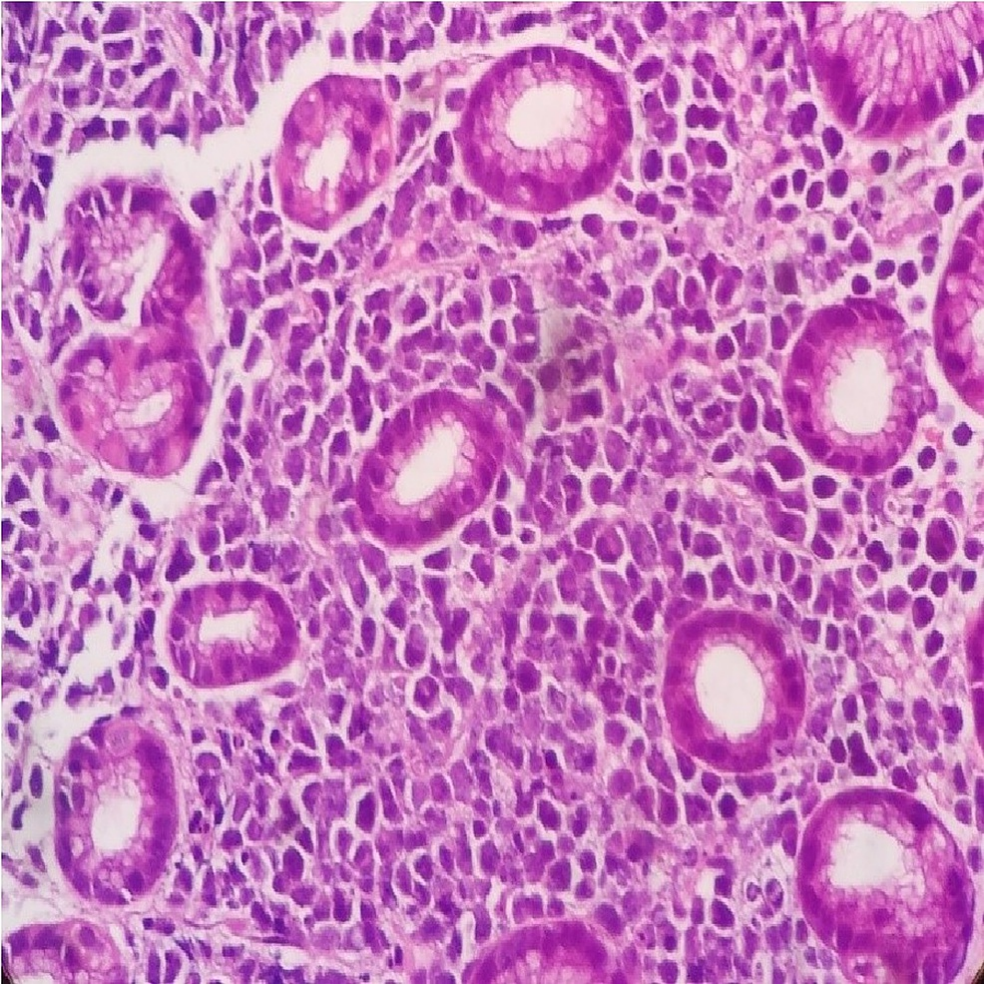
x400 hematoxylin and eosin stain showing gastric mucosa diffusely infiltrated by large cells with varied morphology

**Figure 5:**
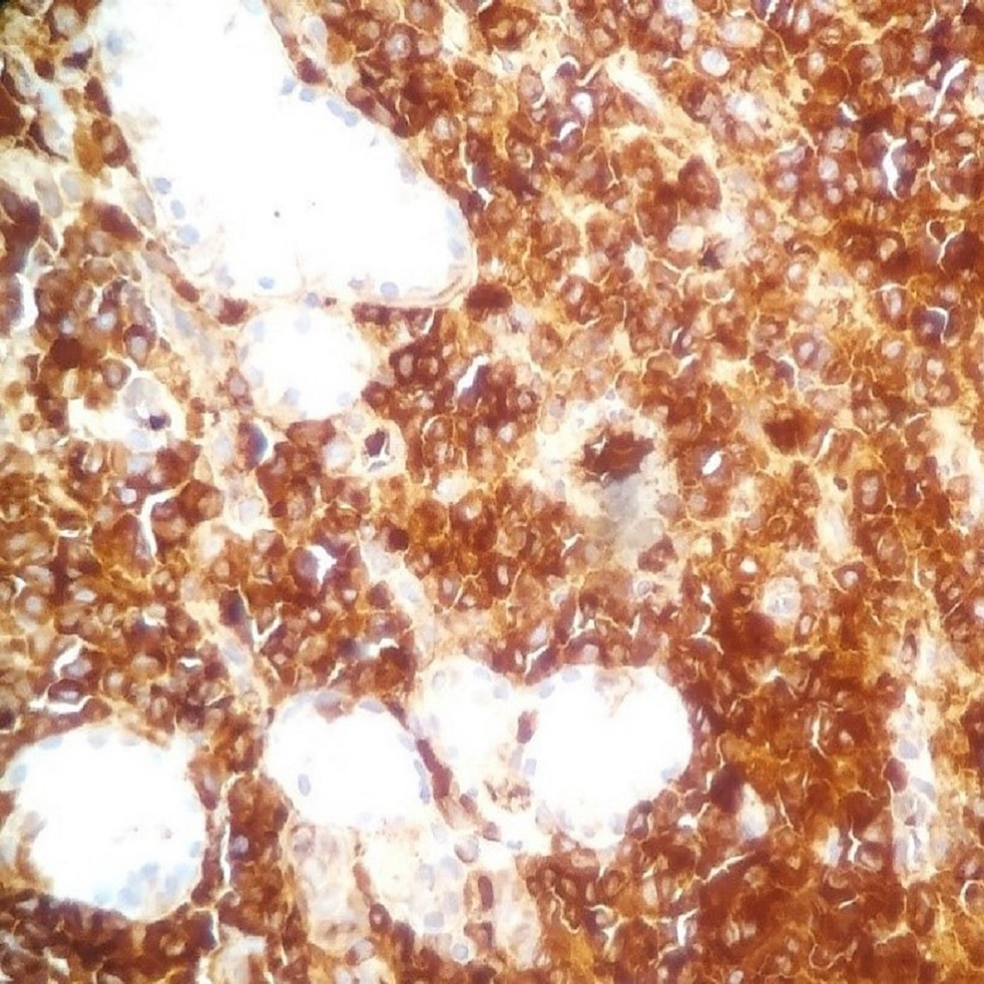
x400 immunohistochemical stain on gastric biopsy with anti-CD38 antibody

**Figure 6:**
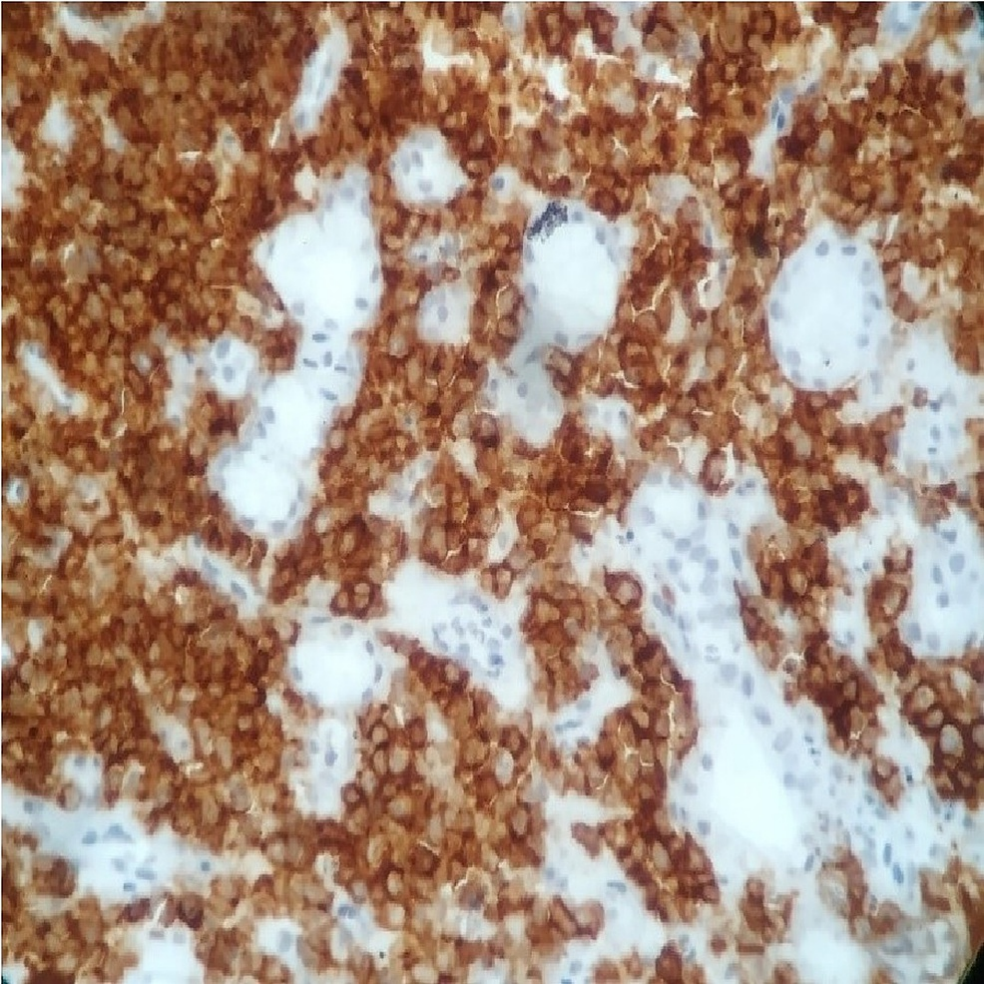
x400 immunohistochemical stain with anti-CD138 antibody

**Figure 7:**
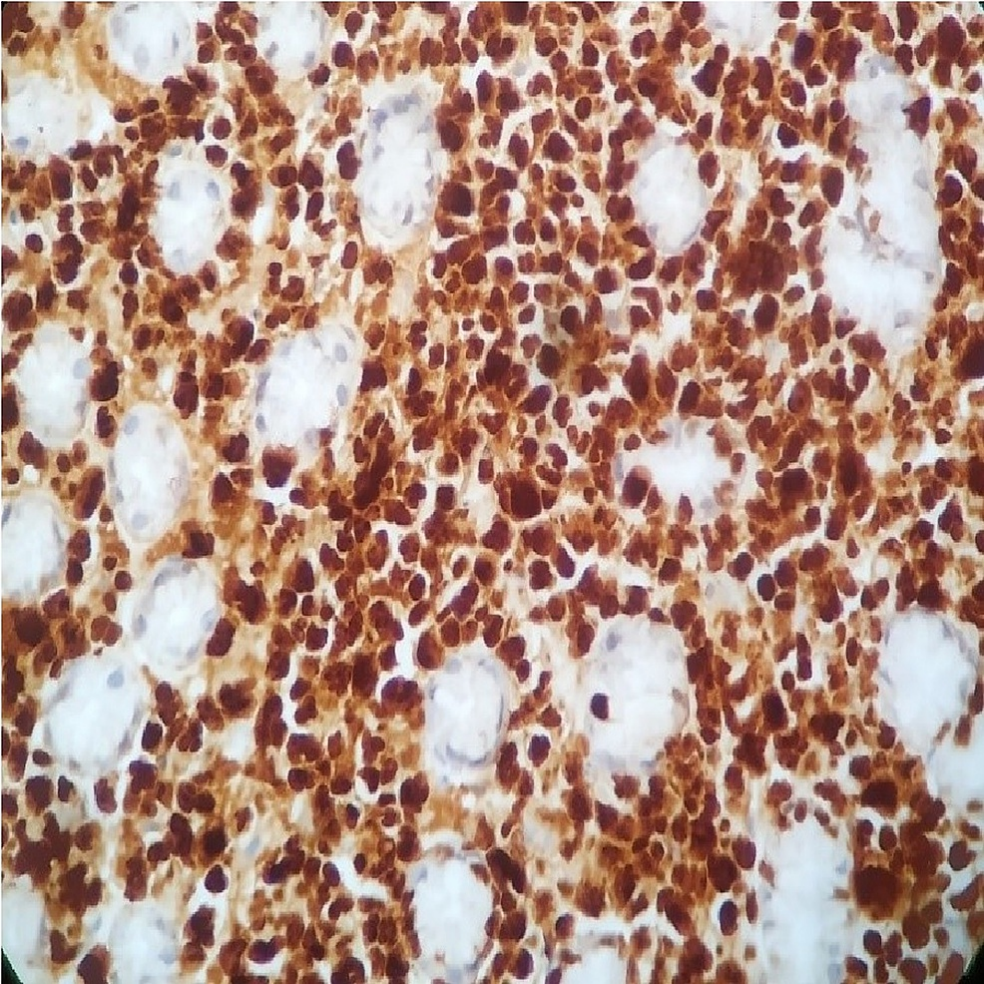
x400 immunohistochemical stain with anti-MUM1 antibody

**Figure 8:**
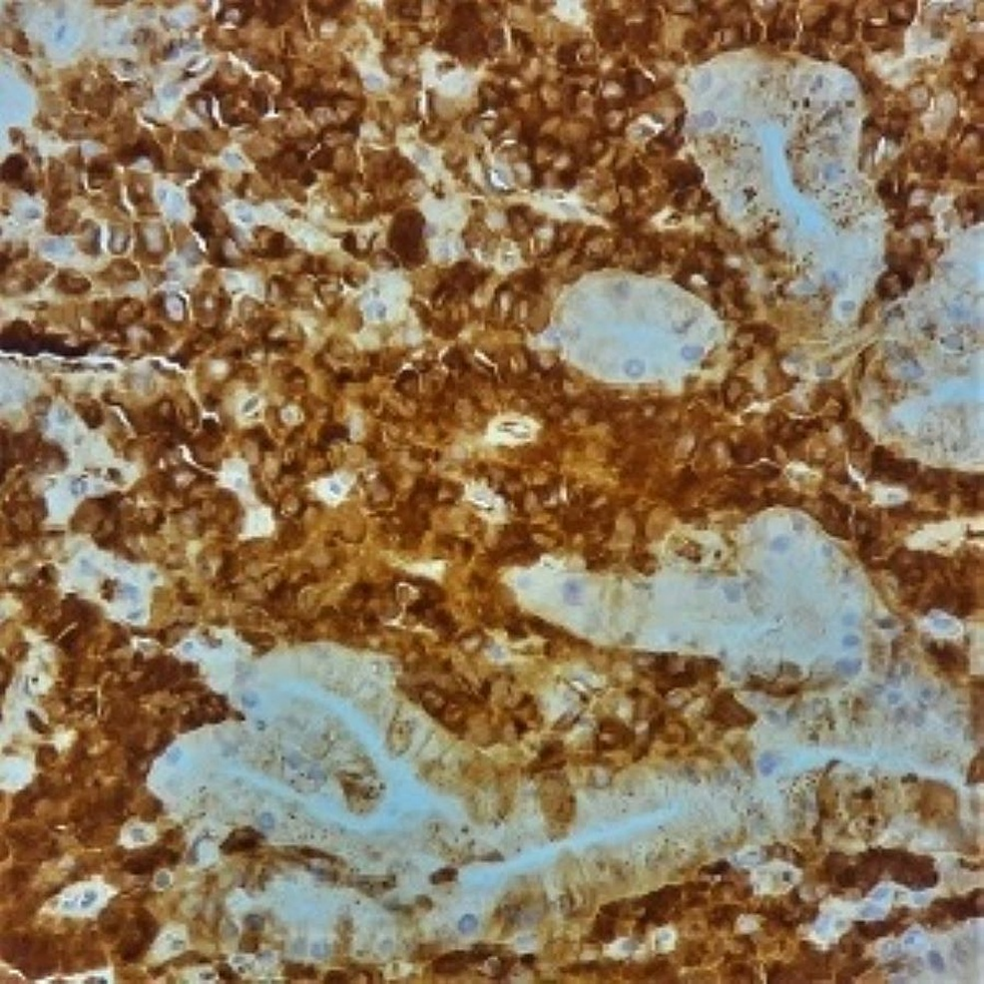
x400 immunohistochemical stain with anti-Kappa antibody

**Figure 9:**
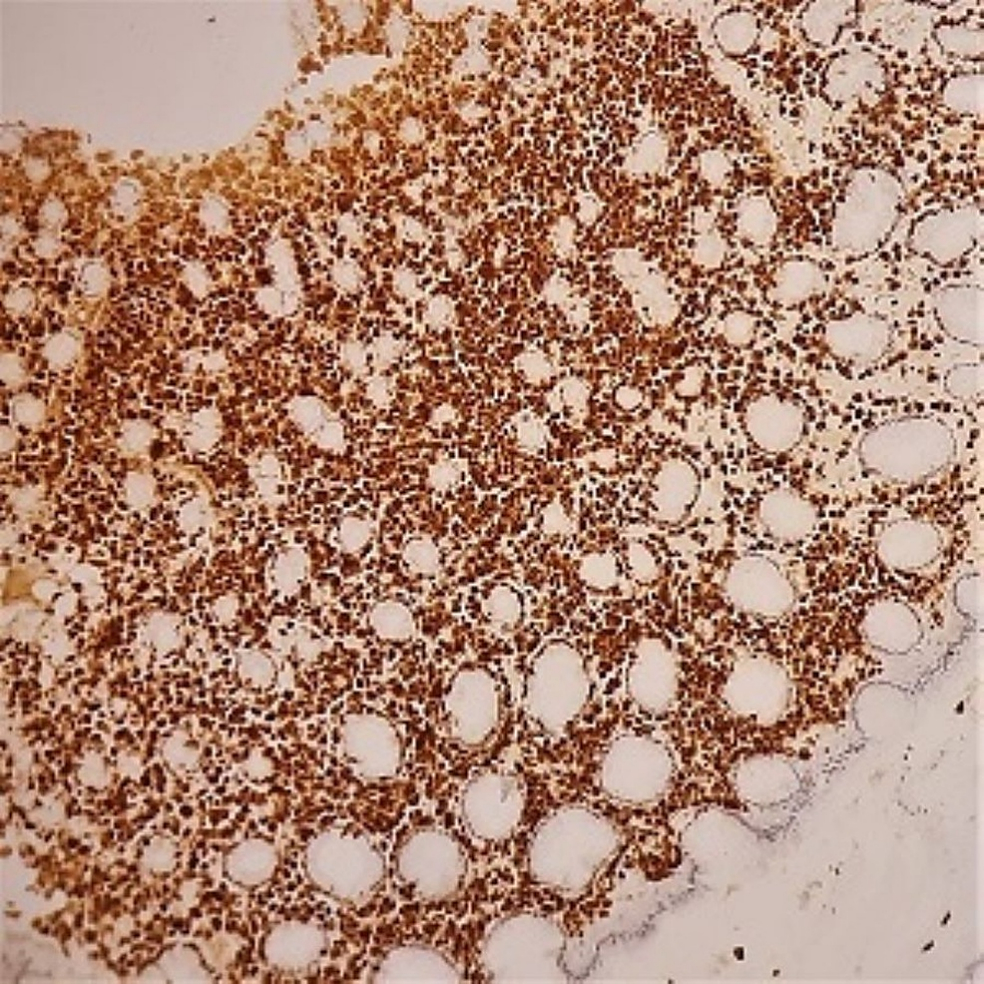
x100 immunohistochemical stain with Ki-67 antibody

The histopathological section from the stomach biopsy showed gastric mucosa infiltrated by a poorly differentiated tumor (Figure 4). These cells had a moderate amount of cytoplasm with varied morphology and a prominent nucleolus in some of them. Many apoptotic bodies were noted. The morphological findings on biopsy are well-correlated with those in the fluid smears. Hence, a high-grade non-Hodgkin lymphoma was suggested and immunohistochemical stains were performed for further categorization. Immunohistochemistry for LCA, Cytokeratin, CD3, and CD20 was negative. The antibodies used in the second panel, which showed a positive reaction were CD38 (Figure 5), CD138 (Figure 6), MUM 1 (Figure 7), and Kappa (Figure 8). CD79a, CD56, CD30, CD5, CD7, ALK 1, and EMA were proved to be negative. The Ki-67 proliferation index was 95% (Figure 9). Finally, a diagnosis of gastric plasmablastic lymphoma was given. Subsequently, bone marrow involvement was seen with 60% plasmacytoid cells (CD38, CD138, and Kappa positive). The patient died within one month of diagnosis.

## Discussion

The suggested cell of origin of this rare aggressive lymphoma is the plasmablast, which is an activated B-lymphocyte that undergoes somatic hypermutation and class switching recombination [7]. Immunophenotypical characteristics of plasmablastic lymphomas are positivity for CD38, CD138, MUM1, MYC, Blimp 1, XBP1, and variable positivity for CD45, CD79a, and EMA. They usually do not stain for B-cell markers like CD20 and PAX5. According to Laurent et al., 60%-70% of cases show positivity for PD-L1 and hence should also be included in the testing panel of antibodies [8].

The closest differential diagnoses of plasmablastic lymphomas are plasmablastic myeloma, diffuse large B-cell lymphoma (plasmablastic type), and ALK-positive large B-cell lymphomas. Immunohistochemical stains aid in the diagnosis and are of help in ruling out diffuse large B-cell lymphoma (DLBCL) and ALK-positive B-cell lymphomas. But the distinction from myelomas is very challenging due to their overlapping immunophenotypes. Both the entities are negative for LCA and stain positively for CD38, CD138, and MUM1. There are few instances in literature where extraoral plasmablastic lymphomas have shown positivity for CD56 and CD79a. However, these findings are quite variable. A high Ki-67 index of more than 80% is almost always documented in plasmablastic lymphomas. Moreover, end-organ damage is seen in patients with multiple myeloma, including hypercalcemia and renal failure. Hence, a complete clinicoradiological correlation is necessary to exclude the diagnosis of multiple myeloma.

In the present case, the morphological diagnostic considerations for lymphoma were DLBCL (plasmablastic type), anaplastic large cell lymphoma (ALCL), and plasmablastic lymphoma. The cytological smears showed a very pleomorphic picture with many cells having a nondescript morphology apart from the ones showing plasmacytoid morphology. There were cells with indented and multilobated nuclei, hence ALCL was also considered. CD20 negativity leads to the exclusion of DLBCL (plasmablastic type). While negative staining for CD3, CD5, CD7, CD30, EMA, and ALK1-excluded ALCL. The absence of any end-organ damage and a high Ki-67 proliferation index favored a diagnosis of plasmablastic lymphoma over myeloma. We did not get any description of indented or multilobated nuclei along with plasmablastic morphology in plasmablastic lymphomas after an extensive literature search. This is a unique feature in our case.

While the association of plasmablastic lymphomas with Epstein Barr virus (EBV) in immunodeficient states (like HIV) is well-documented, its association in immunocompetent patients is suggested to be seen in 50% of cases [9]. However, the impact of the EBV association on overall prognosis is still debatable. In the present case, an in-situ hybridization test for EBV-encoded RNA (EBER) could not be performed, which is one of the limitations of this case.

The commonest cytogenetic abnormality observed in plasmablastic lymphomas is the translocation of Myc with IgH and the amplification of Myc [10].

The preferred chemotherapeutic regimens are hyperfractionated cyclophosphamide, vincristine, doxorubicin, and dexamethasone alternating with methotrexate and cytarabine [9]. Agents that are used in the treatment of plasma cell myeloma (like bortezomib and lenalidomide) have also been tried in patients of plasmablastic lymphoma due to the evidence of plasmacytic differentiation in these lymphomas.

The prognosis of these patients is known to be poor with a median survival of nine to 12 months [2]. The present case also subsequently showed bone marrow involvement with 60% plasmacytoid cells (CD38, CD138, and Kappa positive). The patient died within one month of diagnosis.

## Conclusions

The rarity of plasmablastic lymphomas especially in immunocompetent persons makes awareness of this entity very important. Appropriate clinicoradiological and immunohistochemical workup will help in clinching the diagnosis. This case further highlights unique cytomorphological features, which can be seen along with the established morphological features. Although the treatment regimens haven’t been universalized to date, early diagnosis will pave the way for newer effective treatment strategies.
